# A Reasonable Course to Pursue in the Treatment of Pulpless Teeth*We are reprinting this editorial from the November *Pacific Dental Gazette*, as we printed the editorial criticized in our last issue.

**Published:** 1917-12

**Authors:** Julio Endelman


### "A REASONABLE COURSE TO PURSUE IN THE TREATMENT OF PULPLESS TEETH" *

BY JULIO ENDELMAN, D.D.S.

The editorial in the October issue of the *Journal of the National Dental Association* under the above caption is probably being read at the present time by the thousands of dentists who, by reason of their local society affiliations, are *ipso facto* subscribers to that publication. And where is the dentist—we ask—who would not be attracted by so alluring a promise as that implied in the title of the editorial in question? No problem in the field of dental surgery is of greater significance than that which has to do with pulp infections, their sequelæ, diagnosis, prognosis and treatment, and no other problem is in as profound a state of confusion, the rosy prospects of our distinguished contemporary to the contrary notwithstanding.

* We are reprinting this editorial from the November *Pacific Dental Gazette,* as we printed the editorial criticized in our last issue.

Several phases of the problem are discussed by the editor of the journal, and with some of his conclusions we are constrained to take issue, particularly so because the evidence he brings forth as a basis for some of the deductions recorded in the said editorial is weak and unsupported by scientific inquiry. To wit, it is stated that "the assumption that infections about the ends of pulpless teeth are blood borne from distant parts of the body is (also) without foundation; first, because routine blood cultures in a large number of cases of individuals who have had *dental abscess infection* (italics ours) have failed to reveal bacteria in the blood stream; second, because the bacteria found in chronic dental granuloma (is a so-called dental granuloma ever an acute process?) is by all cultural methods proven to be the same type of bacteria as that which is found on the surfaces of teeth, and all the facts in the case point to the pulp canal (root canal) and pyorrhea pocket as the ports of entry through which these bacteria enter and effect lodgment in the apical areas."

In our opinion the conclusion that the apical areas of tooth roots can not become infected via the blood stream, in the event of the existence of a locus miniors resistentia, is not proven by the arguments above quoted. There are a number of portals of entry to the body for microorganisms, viz., the respiratory tract especially, the nose, pharynx and tonsils; the saliva, or mucus in which bacteria become entangled and swallowed (Adami); the intestinal tract; the skin and mucous membranes, which are not altogether impervious to bacteria. Organisms which have thus entered the body may give rise to reactions in any point of the body in which a lowered degree of resistance exists. Why should the teeth and supporting tissues be the exception of a thoroughly investigated and many times confirmed pathologic fact? Are the bacteria found in a so-called dental granuloma the same as found on the surfaces of teeth, as claimed by the editor of the journal?

Indeed, such is not the case. Has the spirochete dentium, or the leptothrix buccalis maxima, or the spirillum sputigenum, or the leptothrix innominata—organisms commonly found on the surfaces of teeth, ever been detected in these granulomata? And again, does it necessarily follow that because no cultures are obtainable in all instances from dental granulomata, that such granulomata bear no pathologic relation to the microorganisms which we know are their cause? It is the same type of argument as offered in the commentaries by the editor of the *Journal of the N. D. A.* If the bacteria (streptococci) which we know are mainly responsible for chronic infections on the roots of teeth can not be cultured from the blood of the same individual, why should it follow that infection of the apical area can not occur hematogenically from other regions of the body? The streptococcus viridans is of low virulence and its presence in the blood is of short duration, the organisms which find a suitable environment in any area of lowered vitality, being the only survivors, and those which continue in the blood stream being soon annihilated by the bactericidal elements in that fluid. As a matter of fact bacteria may reach the periapical tissues by way of the circulation and become active in some area thereof, or in some other section of the peridental membrane in which resistance by any cause has been previously lowered. This mode of infection, while not of frequent occurrence, is nevertheless not to be overlooked. There is as much logic in accepting the possibilities of metastatis from chronic infections around or upon the roots of the teeth as there is upon assuming that foci of chronic infection upon areas of the body at remote distances from the jaws and teeth may bring about infection of the peridental membrane. The so-called pericemental abscess first observed by D. D. Smith, and studied micropathologically by Kirk, is one acknowledged source of peridental infection via the blood stream.

The editor of the *Journal,* further in his contribution, refers to the investigations and conclusions of Mayrhofer, the essence of which is that following the treatment of a root-canal and the filling thereof by ordinary methods once the root-canal or canals have been rendered sterile, such sterility or asepsis is not constantly maintained because ''sterilization of extreme root ends had not been accomplished.'' Therefore a root filling of the nature of a varnish suggests itself, and none, so far, can answer the purpose more satisfactorily than Callahan's formula, with the addition of aristol, as suggested by Conzett. With the editor's therapeutic conclusions we are in absolute accord, but with the arguments leading to his conclusions, we are not altogether satisfied, because they only tell a minor share of the story. The dentinal tubules on the pulp side, excepting perhaps in late middle life or old age, are of a diameter such as to permit the entrance therein of most bacterial forms concerned in pulp and pericemental infection, and unquestionably some of these bacteria, especially when protected by the products of their activity in the medium in which they are housed, are not affected by the therapeutic agents commonly employed in the effort to eradicate root-canal infections. Further, why should the infection stop just at the ''root tips;'' is there any barrier to their further penetration beyond the root tips, and into the cancellated osseous substance of the jaw? The cause of failure, even though the apical foramen and tubuli be impermeably sealed, is to be found in just that contingency, one of rather frequent occurrence, and of infrequent consideration. The examination of hundreds of ''root tips,'' or thousands for that matter, by Hartzell and Henrici, as quoted in the editorial under analysis, is a proof of questionable and debatable value, from the standpoint of the conclusion that the infection is confined to the ''root tips'' and not beyond there. Therefore, the conclusion that ''the problem of successfully sterilizing and filling root-canals, is to be solved by adopting methods that effectively destroy the bacteria in the tips of the canal as well as in the pulp chamber'' not being based upon a correct interpretation of the pathologic processes in chronic periapical affections falls short of the mark, adds nothing to that which everybody already knows and gets us nowhere nearer the solution of the problem as previously understood.

We also must disagree with the editor of the *Journal of the National Dental Association,* although we are open to conviction through scientifically obtained data, with the statement that the accumulating evidence of the past two years shows conclusively that it is possible to open the root tips of canals, sterilize and fill them and even permit a quantity of chloro-percha to extrude through the root and without permanent damage to the tissues beyond, or ''that the temporary damage wrought by the use of powerful antiseptics or the extrusion of root-canal filling material when sterile is rapidly repaired without permanent damage to the tissues and the teeth so treated remain in a safe and healthy condition.''

We should like to ask what is meant by ''the accumulated evidence;'' also for the data on these extrusions, viz.: the length of the extended filling material for each case observed by the editor or any other investigator and the length of time elapsed between the filling of the root-canal and the time of observation, six months, one, two or more years after the operation was performed. The mere statement that such a method or methods can be practiced with impunity in all cases and that on the same basis concentrations of the powerful antiseptics can be used in root-canal therapeutics, proves nothing, especially in view of the fact that the conclusions of others, the writer included, based upon actual clinical observations, are not in harmony therewith.

It is to be greatly deplored that the representative periodical of dentistry should find it advisable to scatter information which may lead to serious complications, if heeded without proper reservations. The preceding statements in regard to extruded, why not say intruded, root-canal fillings and powerful antiseptics may be correct and true in some cases, in a large number of cases, in a majority of cases, but certainly not, and we most emphatically insist, in all cases. It is contrary to all accepted principles of physiology and pathology; it is dangerous teaching; it is poor dentistry. Microscopic sections of teeth with their investing tissues *in situ* is the strongest argument against permitting any foreign substance to protrude into the peri-apical tissues.

The method of treating infected root-canals, which the editor mentions as the grand finale of his article, is the one credited to Hartzell and Henrici, and which these investigators have evidently found to be satisfactory. It consists of the following steps:

"First, the dead pulps were removed with barbed broaches and dioxogen.

"Second, the pulp canals were treated by churning into them Schreider's sodium and potassium in order to sterilize and dissolve the fatty material.

"Third, the root-canals were washed out with alcohol in order to free them from the soapy sodium potassium hydrate.

"Fourth, the canals were flushed with C. P. sulfuric acid.

"Fifth, the canals were dried out with dry cotton.

"Sixth, the canals were filled by the Callahan method.

"Seventh, was the process of apecoectomy."

Nothing else remains now to be added to this method of successfully treating root-canals, unless it be the extraction of the offending organ. It would be interesting to know what scientific reasons could be advanced in support of the adoption of a method of treatment, which smacks of seventeenth century empiricism.

Oh, Science—what sins are not committed in thy name!

